# COFs in the Game: Harvest Water to Sustain

**DOI:** 10.1021/acscentsci.5c00357

**Published:** 2025-03-07

**Authors:** Grace C. Thaggard, Natalia B. Shustova

**Affiliations:** 2629 University of South Carolina, Department of Chemistry and Biochemistry, 631 Sumter Street, Columbia, South Carolina 29208, United States

## Abstract

Breakthrough covalent-organic frameworks can harvest water directly from air at record-low humidity, paving the way for clean drinking water in arid regions.

## Graphical abstract

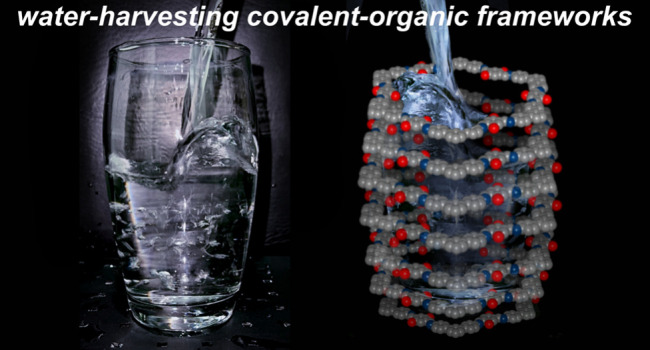

On a scorching morning in the Namib Desert, where rain is a rare miracle and the sun rules the sky, a tiny beetle performs a life-saving ritual. It climbs to the top of a dune, lifts its body into the air, and waits. In the stillness, something remarkable happens—tiny water droplets, invisible to the naked eye, begin to cling to the rough surface of its shell. Slowly, they merge, forming precious beads of liquid that trickle down the beetle’s body and into its mouth. In one of the driest places on Earth, this ingenious creature has learned to drink from thin air.^1^

Meanwhile, thousands of miles away, at the University of California, Berkeley, Prof. Omar Yaghi leads pioneering research on advanced materials called covalent-organic frameworks (COFs) that mimic this beetle’s behavior by efficiently pulling water from the air. For instance, Yaghi, Gagliardi, Sauer, and co-workers have demonstrated that porous materials like COFs can harvest atmospheric water even in the most arid regions on Earth.^2^ This breakthrough technology offers a promising solution to the looming global clean water crisis, which is projected to affect nearly six billion people by 2050.^3^ Thus, what was once an evolutionary survival trick may soon become a revolutionary solution for humanity’s future.

In this issue of *ACS Central Science*, Yaghi’s research team reports a step toward combating global water scarcity by not only setting a new record for atmospheric water-harvesting platforms but also by developing a mathematical model that allows one to predict a material’s onset position of water sorption, a critical parameter for the development of the next-generation water-harvesting devices.

In this issue of *ACS Central Science*, Yaghi’s research team reports a step toward combating global water scarcity by not only setting a new record for atmospheric water-harvesting platforms but also by developing a mathematical model that allows one to predict a material’s onset position of water sorption, a critical parameter for the development of the next-generation water-harvesting devices.^2^

COFs, porous organic materials constructed from molecular building blocks (“linkers”) connected by strong covalent bonds, can possess very high surface areas (e.g., >1000 m^2^ g^–1^), tunable pore sizes, broad thermal and chemical stability, and sorption properties which can be synthetically programmed through a nearly unlimited choice of organic linkers.^4–9^ All of the mentioned properties allow for the design of materials with high water capacities while remaining stable toward adsorbed water and the high temperatures experienced in dry climates.

**1:**
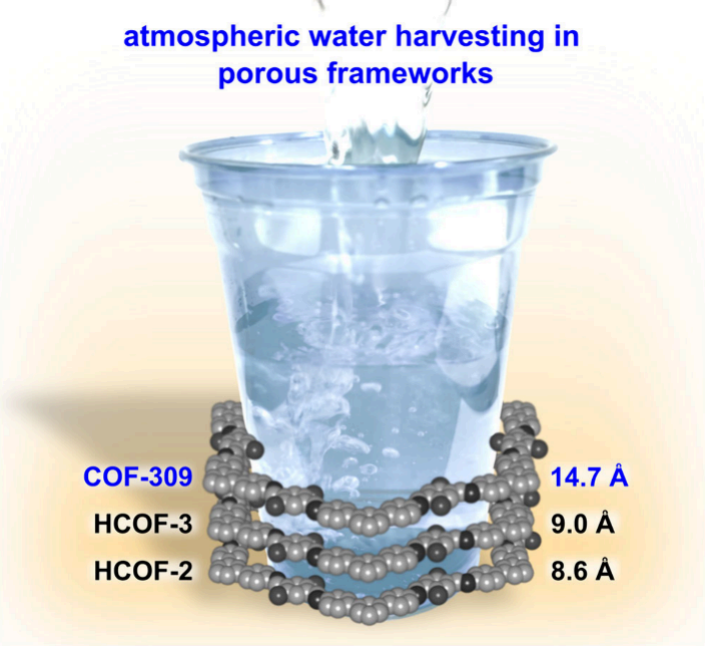
Schematic representation of a porous covalent-organic framework “cup” being filled with water from the atmosphere. Variations in organic linkers and pore sizes can tune the material’s water sorption properties.

The mechanism of water adsorption in COFs can be visualized as an avalanche, which starts with a single falling stone and is quickly followed by a cascade of rocks, boulders, and debris. In water-harvesting COFs, individual water molecules will diffuse through the porous matrix and bind to adsorptive sites, which are organic linkers possessing polar functional groups to facilitate strong hydrophilic interactions (Figure 2).^2^ The COF’s adsorptive sites are quickly saturated, but water vapor continues to diffuse into the bulk material. Hydrogen bonding interactions between neighboring water molecules in the COF pores allow the pore volume to be filled with a network of water.^2^ At first glance, an assumption could be that the key parameter that dictates the atmospheric water harvesting properties of COFs would be pore size since the majority of water molecules are not directly bound to the COF structure but rather to each other. However, as Yaghi describes in his work, the “onset position,” which is the benchmark described as the relative humidity (RH) at which 50% of the maximum water capacity of the COF can be achieved, is not directly linked to pore size alone, but rather to the spatial density and strength of adsorptive sites.^2^ Just as an avalanche cannot happen without displacement of the first rock, the COF pore volume cannot be filled with water without the initial water molecule binding at adsorptive sites in the framework backbone. Therefore, Yaghi and his team designed a COF with an optimized density of adsorptive sites balanced with pore size to achieve a new record in the field of water-harvesting materials (Figure 2).^2^

**Figure 2:**
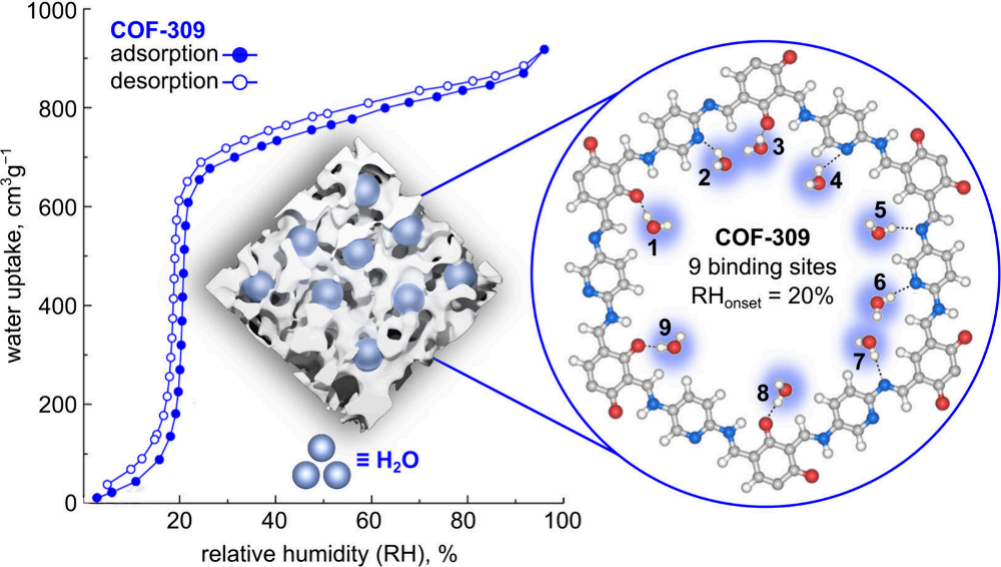
(Left) Water sorption and desorption isotherms of COF-309 demonstrating an onset position at 20% RH. The inset shows a schematic representation of water adsorption in a porous material. (Right) Structure of COF-309 with nine adsorptive sites for water coordination that are highlighted in blue. The gray, red, blue, and white spheres represent carbon, oxygen, nitrogen, and hydrogen atoms, respectively. Reproduced with permission from ref 2. Copyright 2025 American Chemical Society.

COF-309 was not only able to adsorb water but could also release it under mild conditions (60 °C), and therefore, COF-based devices can operate by harvesting water from the atmosphere during the night, while the solar energy during the day initiates the slow release of clean water.

The breakthrough material, called COF-309, possesses nine adsorptive sites per pore formed by the imine and β-ketoenamine linkages in the COF backbone, a Brunauer–Emmett–Teller surface area of 1698 m^2^ g^–1^, and high hydrolytic stability (Figure 2).^2^ As shown in Figure 2, the water sorption isotherm for COF-309 shows a steep step with a record-low onset position of 20% RH and a total water uptake capacity of 0.74 g_water_ g^–1^_COF_ at 25 °C. As a result, the designed COF could be applied for water harvesting in dry climates with low RH. To place the mentioned exciting metrics in context, the majority of water-harvesting COFs reported so far have an onset position in the range of 25–60% RH, which is not compatible with water harvesting in dry regions.^2^ Further, COF-309 was not only able to adsorb water but could also release it under mild conditions (60 °C), and therefore, COF-based devices can operate by harvesting water from the atmosphere during the night, while the solar energy during the day initiates the slow release of clean water.

Overall, the developed model represents a transformative concept that will allow for evaluation of water-harvesting properties across a large class of well-defined hybrid materials. As a result, their model addresses one of the most pressing challenges in the field: quantitative prediction and comparative metrics for any porous frameworks, allowing for data-driven design and analysis of next-generation atmospheric water harvesters.

To build a theoretical model allowing for correlation of framework structure to water sorption onset position (the metric that defines the material’s ability to harvest water), Yaghi and co-workers synthesized two COFs in addition to COF-309 with varying pore sizes and number of adsorptive sites. By evaluating the onset position of three different frameworks, Yaghi and his team developed a parameter called the hydrophilicity index, which relates a material’s water sorption onset position with the thermodynamic strength and spatial density of adsorptive sites. Overall, the developed model represents a transformative concept that will allow for evaluation of water-harvesting properties across a large class of well-defined hybrid materials. As a result, their model addresses one of the most pressing challenges in the field: quantitative prediction and comparative metrics for any porous frameworks, allowing for data-driven design and analysis of next-generation atmospheric water harvesters.
